# One-year efficacy and safety of everolimus-eluting bioresorbable scaffolds in the setting of acute myocardial infarction

**DOI:** 10.1371/journal.pone.0235673

**Published:** 2020-07-09

**Authors:** Yongcheol Kim, SungA Bae, Myung Ho Jeong, Youngkeun Ahn, Chong Jin Kim, Myeong Chan Cho, Andreas Baumbach, Bill D. Gogas, Spencer B. King, Other Korea Acute Myocardial Infarction Registry Investigators

**Affiliations:** 1 Division of Cardiology, Department of Internal Medicine, Yonsei University College of Medicine and Cardiovascular Center, Yongin Severance Hospital, Yongin, Korea; 2 Division of Cardiology, Chonnam National University Hospital, Gwangju, Korea; 3 Department of Cardiology, Cardiovascular Center, Korea University Anam Hospital, Seoul, Korea; 4 Department of Cardiology, Kyung Hee University Hospital at Gangdong, Seoul, Korea; 5 Department of Cardiology, Chungbuk National University Hospital, Cheongju, Korea; 6 Barts Heart Centre, London and Queen Mary University of London, London, United Kingdom; 7 The Spencer B. King III Catheterization Laboratory, Nanjing First Hospital, Nanjing Medical University, Nanjing, China; 8 Division of Cardiology, Department of Medicine, Emory University School of Medicine, Atlanta, Georgia, United States of America; CVPath Institute Inc., University of Maryland, UNITED STATES

## Abstract

**Background and objectives:**

This study sought to compare clinical outcomes between bioresorbable scaffolds (BRS) and durable polymer everolimus-eluting metallic stents (DP-EES) in patients with acute myocardial infarction (AMI) undergoing successful percutaneous coronary intervention (PCI).

**Methods:**

From March 2016 to October 2017, 952 patients with AMI without cardiogenic shock undergoing successful PCI with BRS (n = 136) or DP-EES (n = 816) were enrolled from a multicenter, observational Korea Acute Myocardial Infarction Registry.

**Results:**

In the crude population, there was no significant difference in the 1-year rate of device-oriented composite endpoint (DOCE) and device thrombosis between the BRS and DP-EES groups (2.2% vs. 4.8%, hazard ratio [HR] 0.43, 95% confidence interval [CI] 0.13–1.41, p = 0.163; 0.7% vs. 0.5%, HR 1.49, 95% CI 0.16–13.4, p = 0.719, respectively). BRS implantation was opted in younger patients (53.7 vs. 62.6 years, p < 0.001) with low-risk profiles, and intravascular image-guided PCI was more preferred in the BRS group (60.3% vs. 27.2%, p < 0.001).

**Conclusions:**

At 1-year follow-up, no differences in the rate of DOCE and device thrombosis were observed between patients with AMI treated with BRS and those treated with DP-EES. Our data suggest that imaging-guided BRS implantation in young patients with low risk profiles could be a reasonable strategy in the setting of AMI.

## Introduction

Percutaneous coronary intervention (PCI) with new-generation metallic drug-eluting stents (DES), as compared with bare-metal stents (BMS) and first-generation metallic DES, evidently improved clinical outcomes in patients with ischemic heart disease [1–3]. However, the new-generation metallic DES may have long-term limitations induced by the permanent presence of foreign material in the coronary artery [4]. Thus, everolimus-eluting bioresorbable scaffolds (BRS) such as Absorb^TM^ (Abbott Vascular, Santa Clara, CA, USA) were designed to overcome the limitations of metallic DES [5]. In the four major randomized trials regarding BRS versus durable polymer everolimus-eluting metallic stents (DP-EES) (XIENCE^®^, Abbott Vascular), the 1-year clinical outcomes of BRS were comparable with those of DP-EES, although there were concerns about increased rates of device thrombosis in the BRS group [5–8]. A recent large-scale randomized trial demonstrated that compared with DP-EES, BRS was associated with a significantly higher incidence of device thrombosis [9]. Further, an analysis of the seven randomized trials comparing BRS with metallic DES showed that compared with DP-EES, BRS had a significantly higher rate of target lesion failure and device thrombosis at the 2-year follow-up [10]. Finally, current guidelines recommend that BRS should not be used in clinical practice outside of clinical studies [11]. Nevertheless, the safety of BRS implantation for acute myocardial infarction (AMI) patients has been demonstrated in two randomized studies; no significant differences in the incidence of adverse outcomes and device thrombosis were observed between the BRS and DP-EES groups in these studies [12,13]. However, there is a lack of data regarding clinical outcomes in AMI patients who undergo BRS implantation in a real world setting.

Therefore, we sought to compare the clinical outcomes between patients with AMI undergoing successful PCI with BRS and DP-EES, using a nationwide, multicenter registry for AMI.

## Material and methods

The study population was selected from the Korea Acute Myocardial Infarction Registry (KAMIR) database. The KAMIR is the first nationwide, multicenter, observational registry of patients with AMI in Korea, and it reflects “real-world” treatment practice and outcome in patients with AMI. AMI was diagnosed when there was an increased level of cardiac-specific biomarkers, such as troponin I/T or creatinine kinase-MB, with at least one value above the 99^th^ percentile upper reference limit and with at least 1 of the following: symptom of myocardial ischemia, new significant ST-segment-T wave changes, new left bundle branch block, or pathologic Q waves in two contiguous leads on a 12-lead electrocardiogram, and imaging evidence of new loss of viable myocardium or new regional wall motion abnormality [14].

The study protocol was approved by the ethics committee at each participating center, and the principles of the Declaration of Helsinki were followed (approval number: CNUH-2016-075). Written informed consent was obtained from each patient. If patients were unable to provide consent at the time of presentation, informed consent was obtained from their relative or legal representative.

**Fig 1:**
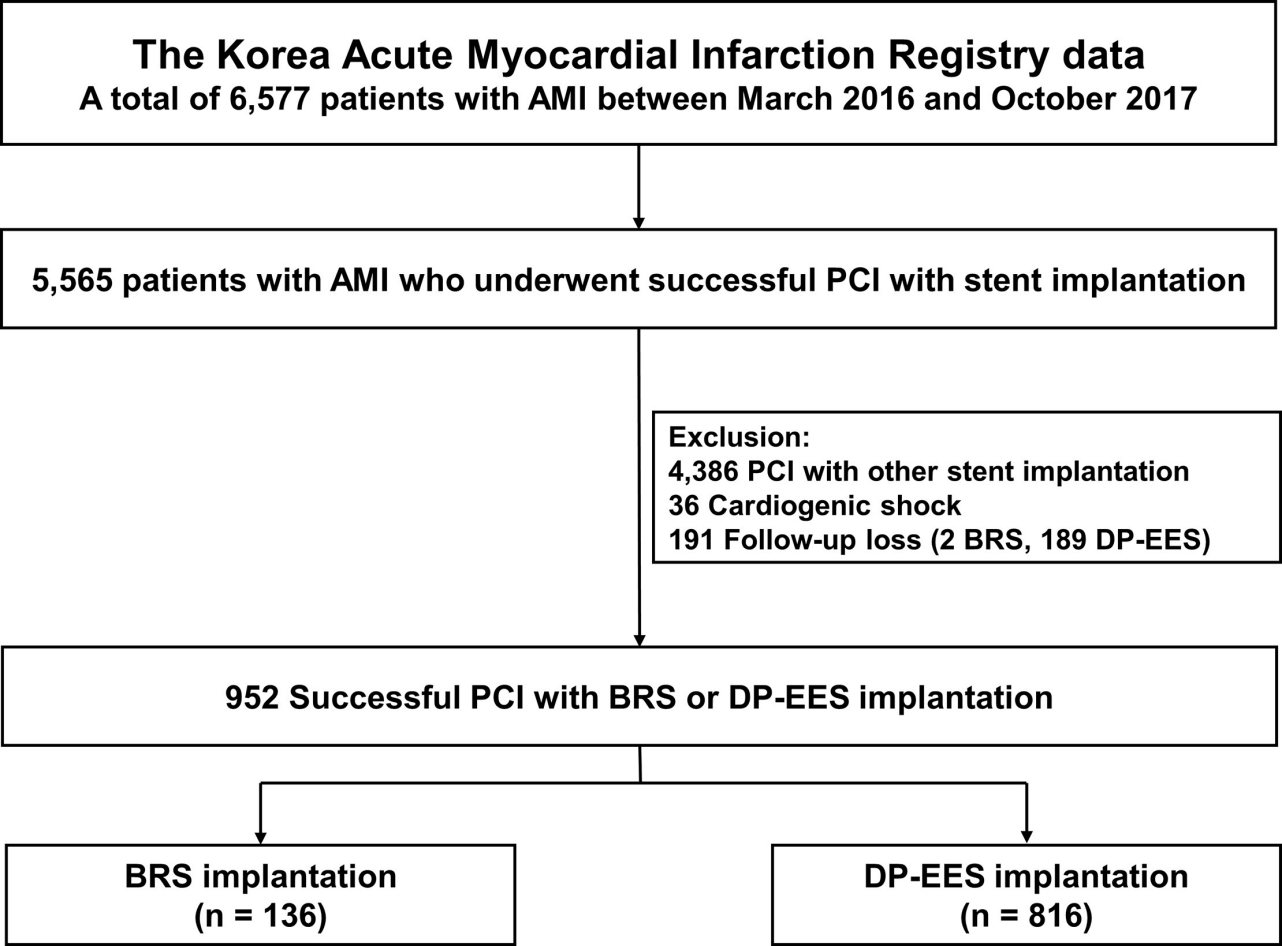
Study flowchart.

Among the 6,970 patients enrolled in KAMIR between March 2016 and October 2017, patients who underwent successful PCI with BRS or DP-EES were selected. Patients who presented with cardiogenic shock or those who were lost to follow-up within the year preceding the study were excluded. Finally, 952 patients were included in the present study (Fig 1).

The patients were managed as per current standard guidelines. The decision of PCI strategy, including the selection of medication, stent, and vascular access, and use of intravascular imaging guidance, thrombus aspiration, and glycoprotein IIb/IIIa inhibitor, was left to the discretion of the operators.

The primary outcome was device-oriented composite endpoint (DOCE) including cardiac death, target vessel myocardial infarction (TV-MI), and ischemic-driven target lesion revascularization (ID-TLR) at 1 year. The secondary endpoint was patient-oriented composite endpoint (POCE) of all-cause death, all MI, and all revascularization at 1 year. All individual components and device thrombosis (stent/scaffold), defined by the Academic Research Consortium criteria, were also analyzed [15].

### Statistical analysis

All continuous variables were expressed as the mean and standard deviation (SD) or median with interquartile ranges (IQR), when appropriate. All categorical variables were reported as numbers with percentages. The continuous variables were compared using the unpaired t-test or Mann–Whitney U test, as appropriate. The categorical variables were analyzed using the χ^2^ test or Fisher’s exact test. Cumulative event rates were calculated based on Kaplan–Meier censoring estimates, and clinical outcomes between the BRS and DP-EES groups were compared using the log-rank test. As differences in baseline characteristics could significantly affect the outcomes, sensitivity analyses were performed to adjust for confounders as much as possible.

First, the hazard ratios of the unadjusted and adjusted models were calculated using Cox proportional hazard models. The following variables were included in multivariate Cox regression analysis: age ≥60 years, ST-elevation myocardial infarction (STEMI), hypertension, intravascular image-guided PCI, and multi-vessel disease (MVD). C-statistics with 95% confidence intervals (CIs) were calculated to validate the discriminant function of the model.

Second, Cox proportional hazard regression in a propensity-score matched cohort was used. The propensity score was estimated for the choice of BRS using a multivariable logistic regression model that included 24 covariates including age, sex, current smoking, hypertension, diabetes mellitus (DM), estimated glomerular filtration rate (eGFR), left ventricular ejection fraction (LVEF), troponin I, clopidogrel, ticagrelor, angiotensin-converting enzyme inhibitor or angiotension-II receptor blocker, beta-blocker, statin, infarct-related artery, American College of Cardiology/American Heart Association B2/C lesion, MVD, vascular access, intravascular image-guided PCI including intravascular ultrasound (IVUS) and optical coherence tomography (OCT), glycoprotein IIb/IIIa inhibitor, pre-PCI Thrombolysis in Myocardial Infarction (TIMI) flow grade 0/1, post-PCI TIMI flow grade 3, mean stent/scaffold diameter, length, and total stent/scaffold number. Propensity score matching yielded 95 patients in the BRS group and 205 control subjects in the DP-EES group. The C-statistic of the logistic regression model for propensity score matching was 0.655. To assess the efficacy of the propensity score model, the standardized differences for each covariate between the groups were calculated. A close to % absolute standard difference for a covariate indicated absence of residual bias, whereas a value within 20% indicated inconsequential residual bias. Balance between the two groups after propensity-score matching was assessed by calculating percent standardized mean differences. Percent standardized mean differences after propensity-score matching were within 20% across all matched covariates, demonstrating successful achievement of balance between the groups. We used a univariate and multivariable Cox proportional hazard model to identify the independent predictor of DOCE and POCE.

All analyses were two-tailed, and a p value < 0.05 was considered to reflect significance. All statistical analyses were performed using R Core Team (2015). R: A language and environment for statistical computing (version 3.3.2, R Foundation for Statistical Computing, Vienna, Austria. URL https://www.R-project.org/)

## Results

### Baseline clinical characteristics

Among the total patient population, 136 patients underwent PCI with BRS and 816 patients underwent PCI with DP-EES. The average patient age was 61.3 ± 11.9 years, and 52.8% of the patients presented with STEMI. Baseline clinical characteristics of the BRS and DP-EES groups are shown in Table 1. The BRS group comprised younger patients (BRS vs. DP-EES, 53.7 ± 10.4 years vs. 62.6 ± 11.6 years; p < 0.001) and more men. The DP-EES group had a higher prevalence of hypertension and DM; however, current smoking was more prevalent in the BRS group. LVEF was more preserved in the BRS group than in the DP-EES group. With regard to medication at discharge, all patients in the BRS group received aspirin, but ticagrelor was more commonly prescribed in this group. In the propensity-matched population, baseline clinical parameters were well-balanced between the groups.

**Table 1:** Baseline clinical characteristics of the study population.

	Overall patients			After propensity-score matching			
	BRS (n = 136)	DP-EES (n = 816)	*p* value	BRS (n = 95)	DP-EES (n = 205)	*p* value	SD (%)
**Demographics**							
Age, years	53.7 ± 10.4	62.6 ± 11.6	<0.001	54.8 ± 10.8	56.2 ± 10.3	0.266	-6.7
Male	123 (90.4)	624 (76.5)	<0.001	85 (89.5)	179 (87.3)	0.731	5.94
BMI, kg/m^2^	24.5 ± 3.0	24.4 ± 3.5	0.801	24.5 ± 3.1	24.3 ± 2.9	0.629	1.2
**Vital sign on admission**							
SBP, mmHg	133.4 ± 26.8	131.5 ± 26.3	0.433	131.8 ± 27.7	133.5 ± 25.6	0.608	-2.45
DBP, mmHg	82.2 ± 18.6	79.3 ± 16.4	0.094	81.0 ± 18.0	82.1 ± 16.3	0.606	-3.67
HR, beat/min	77.2 ± 15.3	78.7 ± 19.2	0.325	77.3 ± 16.0	78.8 ± 18.5	0.471	-0.68
**Initial presentation**							
Diagnosis			0.662			1.000	2.1
STEMI	69 (50.7)	434 (53.2)		45 (47.4)	97 (47.3)		
NSTEMI	67 (49.3)	382 (46.8)		50 (52.6)	108 (52.7)		
Killip class ≥ III	13 (9.6)	82 (10.0)	0.982	8 (8.4)	16 (7.8)	1.000	1.78
**Cardiovascular risk factors**							
Current smoking	80 (58.8)	344 (42.2)	<0.001	51 (53.7)	112 (54.6)	0.977	-0.71
Hypertension	42 (30.9)	390 (47.8)	<0.001	31 (32.6)	76 (37.1)	0.537	-7.19
Diabetes mellitus	21 (15.4)	214 (26.2)	0.010	18 (18.9)	46 (22.4)	0.592	-2.9
Dyslipidemia	14 (10.3)	100 (12.3)	0.610	10 (10.5)	18 (8.8)	0.787	6.33
Familial history of IHD	15 (11.0)	79 (9.7)	0.859	8 (8.4)	21 (10.2)	0.774	-0.56
Prior MI	4 (2.9)	24 (2.9)	1.000	2 (2.1)	8 (3.9)	0.645	-9.31
Prior CHF	3 (2.2)	13 (1.6)	0.877	2 (2.1)	7 (3.4)	0.799	-9.52
Prior CVA	2 (1.5)	44 (5.4)	0.079	2 (2.1)	3 (1.5)	1.000	2.9
**LVEF, %**	55.0 ± 10.3	52.4 ± 10.9	0.010	54.8 ± 10.2	53.0 ± 10.8	0.164	5.85
**eGFR, mL/min/1.73 m**^**2**^	95.8 [80.5–111.8]	83.7 [65.7–104.1]	<0.001	94.9 [80.2–109.0]	86.7 [72.5–107.3]	0.148	1.14
**Peak cardiac enzyme levels**							
Peak CK- MB, ng/ml	80 [22.6–241.0]	56.6 [10.0–191.5]	0.074	80 [23.6–234.8]	47.1 [8.8–165]	0.083	-1.99
Troponin I, ng/ml	23 [6.6–30.0]	30 [11.4–40.0]	<0.001	23 [8.8–31.9]	30 [5.8–40]	0.148	4.3
**Medications at discharge**							
Aspirin	136 (100.0)	809 (99.1)	0.588	95 (100.0)	203 (99.0)	0.839	0.1
Clopidogrel	36 (26.5)	309 (37.9)	0.014	25 (26.3)	56 (27.3)	0.967	-3.17
Ticagrelor	101 (74.3)	482 (59.1)	0.001	72 (75.8)	150 (73.2)	0.734	4.0
Prasugrel	7 (5.1)	61 (7.5)	0.426	6 (6.3)	11 (5.4)	0.950	0.79
ACEI or ARB	116 (85.3)	607 (74.4)	0.008	78 (82.1)	165 (80.5)	0.862	-0.49
Beta-blocker	116 (85.3)	652 (77.9)	0.175	79 (83.2)	172 (83.9)	1.000	0.49
Statin	135 (99.3)	756 (92.6)	0.006	94 (98.9)	199 (97.1)	0.556	9.32
Oral anticoagulant	2 (1.5)	26 (3.2)	0.411	1 (1.1)	3 (1.5)	1.000	0

Values are mean ± SD, median [interquartile range], or n (%).

ACEI, angiotensin-converting enzyme inhibitor; ARB, angiotensin-II receptor blocker; BMI, body mass index; BRS, bioresorbable scaffolds; CHF, congestive heart failure; CK-MB, creatine kinase-myocardial band; CVA, cerebrovascular accident; DBP, diastolic blood pressure; DP-EES, durable polymer everolimus-eluting metallic stents; eGFR, estimated glomerular filtration rate; HR, heart rate; IHD, ischemic heart disease; LVEF, left ventricular ejection fraction; MI, myocardial infarction; SBP, Systolic blood pressure; STEMI, ST-segment elevation myocardial infarction.

### Angiographic and procedural characteristics

The angiographic and procedural characteristics of all patients are presented in Table 2. No patients in the BRS group had the left main coronary artery as the infarct-related artery, whereas 3.7% of patients in the DP-EES group had the left main coronary artery as the culprit lesion. Compared with patients from the BRS group, those from the DP-EES group were more likely to have MVD. The transradial approach, thrombus aspiration, and use of glycoprotein IIb/IIIa inhibitors were more frequent in the BRS group. Moreover, more patients in the BRS group underwent intravascular image-guided PCI (BRS vs. DP-EES, 60.3% vs. 27.5%; p < 0.001), especially under OCT guidance (BRS vs. DP-EES, 36.8% vs. 2.5%). Compared with the DP-EES group, the BRS group showed a larger mean implanted device diameter (BRS vs. DP-EES, 3.3 ± 0.3 mm vs. 3.2 ± 0.4 mm; p < 0.001), shorter device length (BRS vs. DP-EES, 21.4 ± 4.5 mm vs. 25.1 ± 7.1 mm; p < 0.001), and fewer implanted devices (BRS vs. DP-EES, 1.2 ± 0.6 vs. 1.5 ± 0.8; p < 0.001). After propensity score matching, the angiographic and procedural characteristics were well-balanced between the groups.

**Table 2:** Angiographic and procedural characteristics of the study population.

	Overall patients			After propensity-score matching			
	BRS (n = 136)	DP-EES (n = 816)	*p* value	BRS (n = 95)	DP-EES (n = 205)	*p* value	SD (%)
**Lesion profiles**							
Infarct-related artery			0.057			0.991	
LMCA	0 (0)	30 (3.7)		0 (0)	0 (0)		0
LAD	80 (58.8)	407 (49.9)		55 (57.9)	117 (57.1)		-
LCx	19 (14.0)	130 (15.9)		14 (14.7)	31 (15.1)		-1.51
RCA	37 (27.2)	249 (30.5)		26 (27.4)	57 (27.8)		-1.57
ACC/AHA B2/C lesion	121 (89.0)	703 (86.2)	0.449	83 (87.4)	180 (87.8)	1	-3.35
Multi-vessel disease	16 (11.8)	203 (24.9)	0.001	14 (14.7)	36 (17.6)	0.657	-3.8
**Procedural characteristics**							
Transradial approach	116 (85.3)	474 (58.1)	<0.001				-6.91
Intravascular image-guided PCI	82 (60.3)	224 (27.5)	<0.001	44 (46.3)	80 (39.0)	0.286	3.57
IVUS-guided PCI	32 (23.5)	204 (25.0)		28 (29.5)	61 (29.8)		-0.41
OCT-guided PCI	50 (36.8)	20 (2.5)		16 (16.8)	19 (9.3)		3.99
Glycoprotein IIb/IIIa inhibitor	32 (23.5)	107 (13.1)	0.002	19 (20.0)	37 (18.0)	0.807	2.06
Thrombus aspiration	41 (30.1)	110 (13.5)	<0.001	23 (24.2)	40 (19.5)	0.437	7.62
Pre-PCI TIMI flow grade 0/1	54 (39.7)	452 (55.4)	0.001	42 (44.2)	88 (42.9)	0.933	5
Post-PCI TIMI flow grade 3	131 (96.3)	773 (94.7)	0.566	93 (97.9)	200 (97.6)	1	2.79
Mean stent/scaffold diameter, mm	3.3 ± 0.3	3.2 ± 0.4	<0.001	3.3 ± 0.3	3.2 ± 0.4	0.474	1.62
Mean stent/scaffold length, mm	21.4 ± 4.5	25.1 ± 7.1	<0.001	22.0 ± 4.5	22.3 ± 6.4	0.656	4.57
Total stent/scaffold number, n	1.2 ± 0.6	1.5 ± 0.8	<0.001	1.3 ± 0.7	1.3 ± 0.6	0.617	-2.47

Values are mean ± SD or n (%).

ACC/AHA, American College of Cardiology/American Heart Association; BRS, bioresorbable scaffolds; DP-EES, durable polymer everolimus-eluting metallic stents; IVUS, intravascular ultrasound; LAD, Left anterior descending artery; LCx, Left circumflex artery; LMCA, Left main coronary artery OCT, optical coherence tomography; PCI, percutaneous coronary intervention; RCA, right coronary artery; TIMI, Thrombolysis in Myocardial Infarction

### Clinical outcomes according to the device used

**Fig 2:**
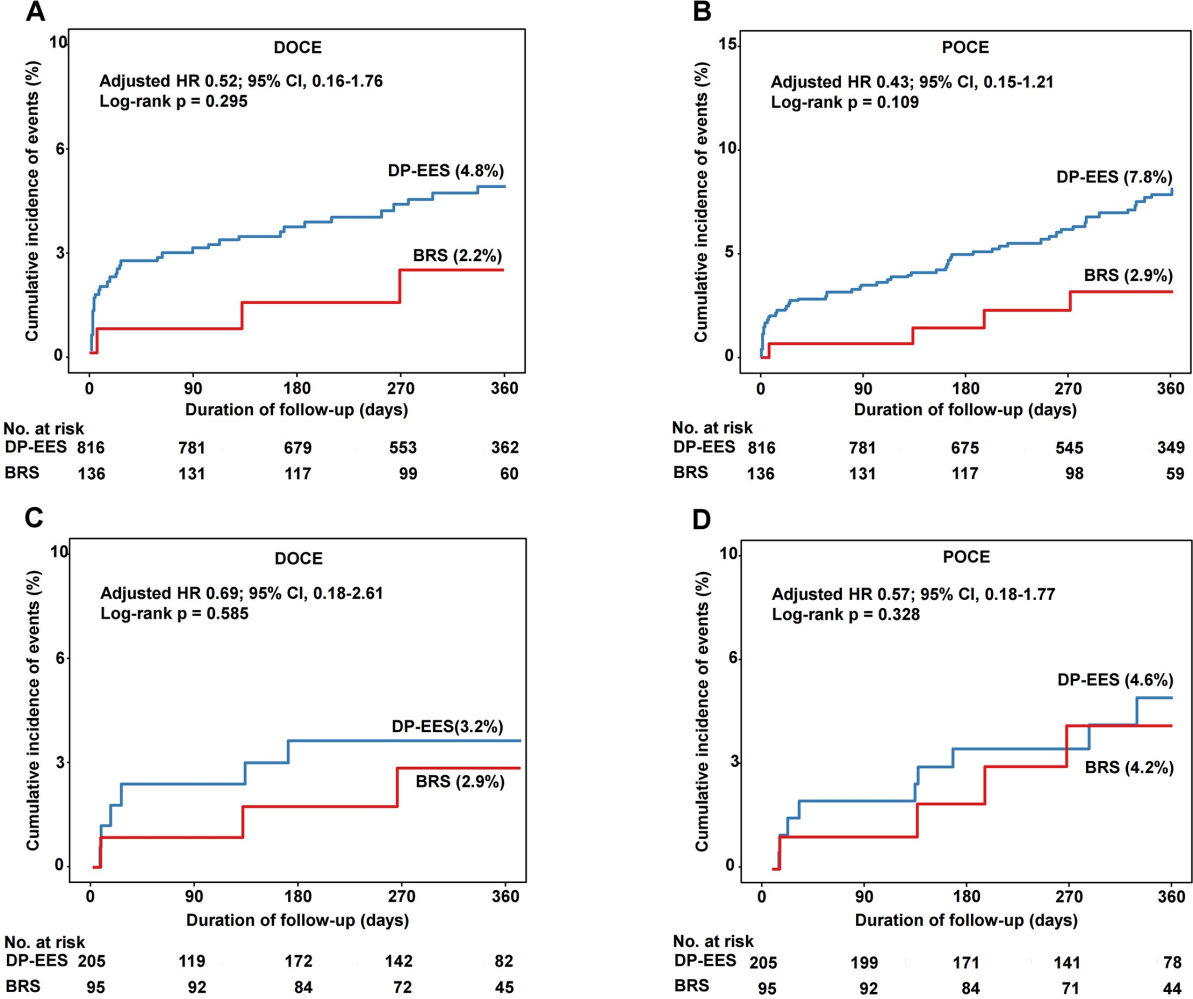
Cumulative incidence of DOCE and POCE in the crude population (A and B) and propensity-matched population (C and D). BRS, bioresorbable scaffold; DP-EES, durable-polymer everolimus-eluting metallic stent; DOCE, device-oriented composite endpoint; POCE, patient-oriented composite endpoint.

In the crude population, there was no significant difference in the 1-year rate of DOCE between the groups (Fig 2). The risk of POCE was significantly lower in the BRS group than in the DP-EES group; however, multivariate adjustment and propensity score matching did not show any difference in the 1-year rate of POCE between the groups (Table 3). There was no significant difference between the groups in the rates of cardiac death, TV-MI, ID-TLR, all death, all MI, and all revascularization. The 1-year rate of device thrombosis did not differ between the groups (Table 3).

**Table 3:** Comparison of 1-year clinical outcomes between the groups.

	BRS (n = 136)	DP-EES (n = 816)	Unadjusted		Adjusted		PS-adjusted	
			HR (95% CI)	*p* value	HR (95% CI)	*p* value	HR (95% CI)	*p* value
DOCE	2.2 (3)	4.8 (39)	0.43 (0.13–1.41)	0.163	0.52 (0.16–1.76)	0.295	0.69 (0.18–2.61)	0.585
Cardiac death	0.7 (1)	2.8 (23)	0.26 (0.04–1.92)	0.187	0.34 (0.04–2.55)	0.293	0.89 (0.09–8.95)	0.928
TV-MI	0.7 (1)	1.3 (11)	0.45 (0.06–3.56)	0.449	0.53 (0.06–4.83)	0.573	0.39 (0.04–3.47)	0.397
ID-TLR	1.5 (2)	1.8 (15)	0.70 (0.16–3.10)	0.636	0.87 (0.18–4.17)	0.863	0.62 (0.12–3.14)	0.563
POCE	2.9 (4)	7.8 (64)	0.36 (0.13–0.98)	0.045	0.43 (0.15–1.21)	0.109	0.57 (0.18–1.77)	0.328
All death	0.7 (1)	3.8 (31)	0.19 (0.03–1.41)	0.105	0.27 (0.04–2.03)	0.204	0.41 (0.04–3.87)	0.435
All MI	1.5 (2)	2.6 (21)	0.51 (0.12–2.19)	0.367	0.63 (0.13–2.95)	0.556	0.58 (0.12–2.85)	0.503
All revascularization	2.2 (3)	3.8 (31)	0.53 (0.16–1.75)	0.297	0.54 (0.16–1.87)	0.330	0.73 (0.19–2.81)	0.650
Device thrombosis (definite/probable)	0.7 (1)	0.5 (4)	1.49 (0.16–13.4)	0.719	1.73 (0.15–19.8)	0.658	1.02 (0.09–11.6)	0.989

Values are % (n) unless otherwise indicated.

BRS, bioresorbable scaffolds; CI, confidence interval; DOCE, device-oriented clinical endpoint; DP-EES, durable polymer everolimus-eluting metallic stents; HR, hazard ratio; ID-TLR, ischemic driven-target lesion revascularization; POCE, patient-oriented clinical endpoint; PS, propensity score; TV-MI, target vessel myocardial infarction

### Independent predictors of DOCE and POCE

A multivariate Cox proportional hazard model revealed independent predictors of the primary and secondary outcomes (Table 4). Age and Killip class ≥ III were the significant and independent predictor of DOCE (HR: 1.03, 95% CI: 1.01–1.06, p = 0.017; HR: 4.90, 95% CI: 2.58–9.31, p < 0.001, respectively) and POCE (HR: 1.03, 95% CI: 1.01–1.06, p = 0.020; HR: 4.20, 95% CI: 2.17–8.14, p < 0.001).

**Table 4:** Independent predictors of clinical outcomes.

	Univariate		Multivariate	
	HR (95% CI)	*P* value	HR (95% CI)	*P* value
**DOCE**				
BRS implantation	0.43 (0.13–1.4)	0.163		
Age ≥ 60 years	1.05 (1.02–1.08)	<0.001	1.03 (1.01–1.06)	0.017
STEMI	1.61 (0.86–3.03)	0.139		
Killip class ≥ III	5.55 (2.94–10.49)	<0.001	4.90 (2.58–9.31)	<0.001
HTN	1.20 (0.65–2.20)	0.557		
LVEF < 50%	1.35 (0.73–2.47)	0.338		
eGFR < 60 mL/min/1.73 m^2^	3.02 (1.62–5.64)	0.001	1.77 (0.89–3.51)	0.101
Image-guided PCI	0.73 (0.37–1.46)	0.376		
Multivessel PCI	0.93 (0.45–1.96)	0.856		
**POCE**				
BRS implantation	0.36 (0.13–0.98)	0.045	0.65 (0.19–2.17)	0.483
Age ≥ 60 years	1.04 (1.02–1.06)	<0.001	1.03 (1.01–1.06)	0.020
STEMI	1.67 (1.01–2.75)	0.044	1.56 (0.83–2.96)	0.170
Killip class ≥ III	4.47 (2.66–7.48)	<0.001	4.20 (2.17–8.14)	<0.001
HTN	1.35 (0.84–2.18)	0.213		
LVEF < 50%	1.53 (0.95–2.47)	0.078		
eGFR < 60 mL/min/1.73 m^2^	1.92 (1.13–3.26)	0.016	1.73 (0.87–3.45)	0.118
Image-guided PCI	0.8 (0.47–1.36)	0.413		
Multivessel PCI	1.04 (0.59–1.82)	0.891		

BRS, bioresorbable scaffolds; CI, confidence interval; DOCE, device-oriented clinical endpoint; eGFR: estimated glomerular filtration rate; HR, hazard ratio; HTN, hypertension; LVEF, left ventricular ejection fraction; PCI, percutaneous coronary intervention; POCE, patient-oriented clinical endpoint; STEMI, ST-segment elevation myocardial infarction.

### Subgroup analysis

**Fig 3:**
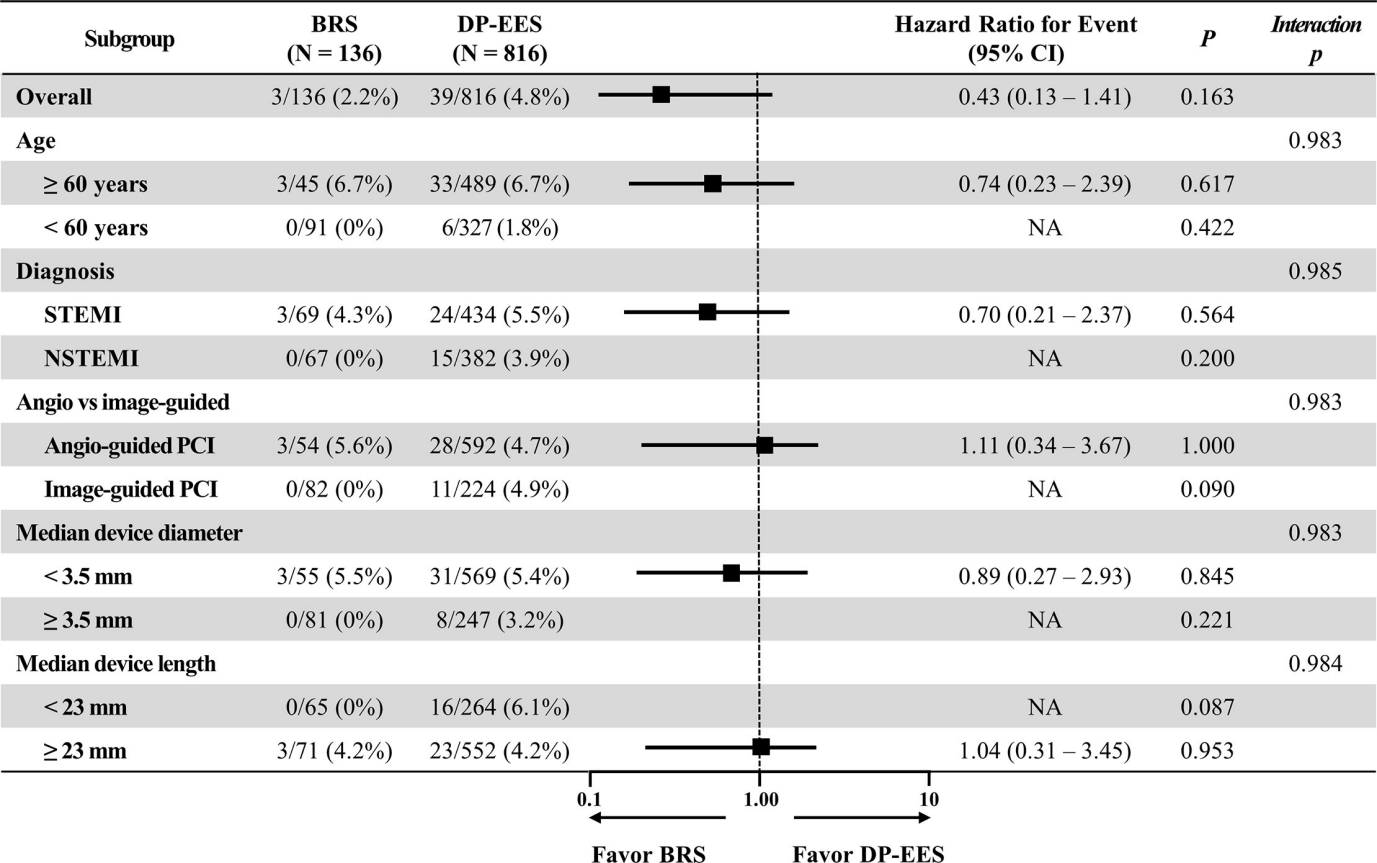
Exploratory subgroup analysis for DOCE. DOCE, device-oriented composite endpoint; NSTEMI, non-ST-elevation myocardial infarction; PCI, percutaneous coronary intervention; STEMI, ST-elevation myocardial infarction.

The subgroup analysis for 1-year clinical outcomes showed no significant difference between BRS and DP-EES implantation across the subgroups (Fig 3). There were no DOCE in young age (< 60 years), non-ST-elevation myocardial infarction (NSTEMI), image-guided PCI, large device diameter (≥ 3.5 mm), and short device length (< 23 mm) groups

## Discussion

**Fig 4:**
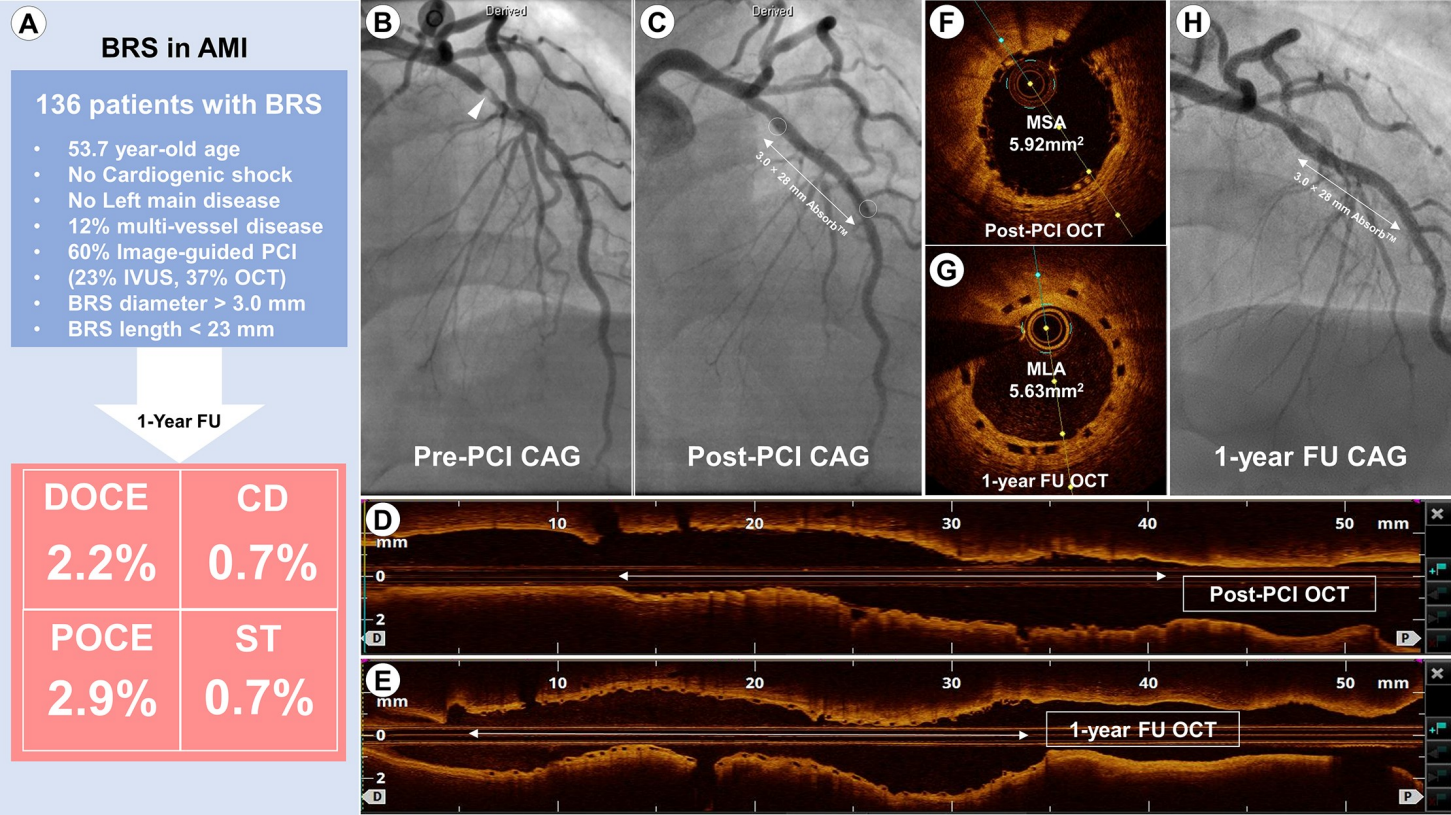
Clinical outcomes of BRS in the setting of AMI and an example of successful OCT-guided BRS implantation in a patient with AMI. (A) The current study showed “real-world” characteristics in patients undergoing BRS implantation and clinical outcomes of BRS and DP-EES were comparable. Angiography in 52-year old woman presenting with NSTEMI (troponin-I = 35.4 ng/mL) showed significant stenosis in the LAD (B, arrowhead). A 3.0 × 28 mm BRS was deployed after pre-dilation with a 3.0 × 15 mm compliance balloon, and post-dilation was achieved with a 3.5 × 12 mm non-compliance balloon at 18 atmosphere (C, white circles indicating radio-opaque markers of BRS). On post-PCI OCT, longitudinal (D) and cross-sectional views (F) showed good scaffold apposition, with good embedment of scaffolds and an MSA of 5.92 mm^2^. One-year follow-up angiography (H) shows the patient with implanted BRS and follow-up OCT (E and G) demonstrated that the scaffolds were well encapsulated by the neointimal tissue.

AMI, acute myocardial infarction; BRS, bioresorbable scaffold; CAG, coronary angiography; CD, cardiac death; DOCE, device-oriented composite endpoint; DP-EES, durable-polymer everolimus-eluting metallic stent; FU, follow-up; IVUS, intravascular ultrasound; LAD, left anterior descending artery; MLA, minimal lumen area; MSA, minimal scaffold area; NSTEMI, non-ST-elevation myocardial infarction; OCT, optical coherence tomography; POCE, patient-oriented composite endpoint; PCI, percutaneous coronary intervention; ST, stent thrombosis.

In the present study, we compared the 1-year clinical outcomes between BRS and DP-EES treatment in patients with AMI using a nationwide, multicenter, registry data. The main findings of our study were as follows (Fig 4): 1) there were no differences in the incidence of DOCE, POCE, or device thrombosis between the BRS and DP-EES groups; 2) compared with DP-EES, BRS was implanted in younger patients with low risk profiles including lower incidence of MVD, no cardiogenic shock, and no left main disease; and 3) in the BRS group, intravascular image-guided PCI, especially, OCT-guided BRS implantation, was performed more often than in the DP-EES group.

The previous two studies that compared BRS with DP-EES in the setting of AMI have reported no differences in clinical outcomes between the BRS and DP-EES groups [12,16]. The first randomized control trial regarding bioresorbable vascular scaffolds in patients with STEMI demonstrated comparably low prevalence of DOCE and definite device thrombosis at 6 months between the BRS and DP-EES groups [12]. In the propensity-score matched study, the BRS and DP-EES groups showed similar rates of 1-year DOCE and device thrombosis [16]. However, compared with the present study in which patients were enrolled from March 2016 to October 2017, these two trials enrolled only patients with STEMI, and the study population was enrolled in the early phase of BRS use (December 2012 to September 2014). Recently, the ISAR-Absorb MI (Intracoronary Scaffold Assessment a Randomized evaluation of Absorb in Myocardial Infarction), a prospective randomized trial comparing clinical outcomes after BRS and DP-EES in patients with AMI, reported comparable 1-year clinical events between the two groups (DOCE: 7.0% [BRS] vs. 6.7% [DP-EES]; POCE; 15.1% [BRS] vs. 14.6% [DP-EES]; device thrombosis: 1.7% [BRS] vs. 2.3% [DP-EES], respectively) [13]. The present study of patients not enrolled in a randomized trial also showed no significant differences in the risk of 1-year DOCE and device thrombosis between the groups. Compared with the ISAR-Absorb MI, however, the BRS group in this study had a relatively lower incidence of clinical events (DOCE: 2.2% vs. 7.0%; POCE: 2.9% vs. 15.1%; device thrombosis: 0.7% vs. 1.7%, respectively [present study vs. ISAR-Absorb MI]). These differences could be explained by different characteristics of patients in this study. First, the mean age of enrolled patients was lower in this study than in other AMI-BRS studies [12,13,16]. Patients enrolled in this study were more than 10 years younger than the average Korean patients with AMI [17]. Young patients have less calcified plaque and diffuse atherosclerosis in the coronary lesion. Moreover, the most common plaque morphology in young patients is soft fibroatheroma [18]. Fibroatheroma enables proper embedment and less protrusion of the scaffolds into the lumen, which could produce better clinical outcomes [19,20]. Therefore, such patients might be ideal candidates for BRS implantation, and their inclusion in this study may have resulted in a low prevalence of adverse outcomes. Second, MVD is significantly associated with increased adverse prognosis [21,22]. Although 40% of patients from the BRS group had MVD in the ISAR-Absorb MI trial, the prevalence of MVD was about 12% in this study. Moreover, there was no left main disease as an infarct-related artery in the BRS group. Regarding device profiles, shorter device length and fewer implanted devices were observed in the BRS group than in the DP-EES group, which might represent that lesions have less complexity in the BRS group. Thus, less severe angiographic lesion characteristics in patients receiving BRS could account for the good prognosis in the present study.

Interestingly, 60% of patients receiving BRS underwent intravascular image-guided procedures; in particular, OCT-guided PCI was performed in 36.8% of patients in the BRS group compared with only 2.3% of patients in the DP-EES group. Considering the annual trends of OCT-guided PCI in Korean patients with AMI, the rate of OCT-guided PCI was high in the BRS group in this study [23]. The superior resolution (10 μm) of OCT mitigates device failure by adequate lesion preparation, appropriate choice of diameter and length of BRS, and full expansion of BRS (Fig 4) [24]. Therefore, intravascular image-guided PCI may have been helpful for BRS optimization to prevent adverse clinical events including device thrombosis.

In our subgroup analysis for 1-year clinical outcomes, patients aged older than 60 years, those presenting with STEMI, or those underwent device implantation by angiography guidance showed similar clinical outcomes in both groups. Interestingly, our study showed no adverse events at 1 year in patients aged less than 60 years, NSTEMI patients, and patients who had image-guided PCI in the BRS group. However, meta-analysis demonstrated that BRS had worse clinical outcomes including device thrombosis, at the 2-year follow-up [10]. Thus, 2-year follow-up coronary angiography or coronary computed tomography angiography might be considered to detect device failure, especially in patients older than 60 years, STEMI patients, and patients underwent angiography-guided PCI, although there were no guidelines of follow-up for patients with BRS implantation. Regarding the subgroup analysis of BRS profiles, there was no DOCE in patients receiving large diameter (≥ 3.5 mm) or short length (< 23 mm) of BRS. In other words, the effect of BRS for anatomically complex lesions in prior BRS clinical studies [10,25]. In the previous report, the scaffold design (i.e. thicker strut with relatively lower radial strength versus metallic stent) was considered as one of the reasons. Thus, the innovation of material design with reduced strut thickness maintaining radial strength is desired for the next generation BRS.

There were several limitations in the present study. First, this study has an inherent limitation owing to observational data and small study population, despite the use of a large multicenter registry. Compared with DP-EES, BRS was implanted in younger patients with simpler lesions, as the selection of the device used in this study was left to the discretion of the operators. Further, patients with cardiogenic shock were excluded from this study as no patient in the BRS group was in a state of cardiogenic shock. As a result, only 136 patients underwent PCI with BRS, and this small sample size limits the value of careful statistical analysis, including propensity-score matching adjustment. Second, we could not evaluate BRS-specific protocols, the so-called Pre-dilation, Sizing, Post-dilation (PSP) strategy, which has been significantly associated with reduced rates of device failure [26]. Since the PSP strategy was first introduced by a group of European experts in May 2015, multicenter registry data demonstrated the effectiveness of the PSP strategy to reduce scaffold thrombosis in March 2016 [27,28]. Therefore, operators may have applied the PSP strategy to most patients in this study, as patients were enrolled between March 2016 and October 2017. Third, although the plaque characteristics of young patients and a high proportion of intravascular image-guided PCI procedures may have led to better clinical outcomes in this study, a detailed analysis of intravascular images including IVUS and OCT was not performed. Despite these limitations, our study showed “real-world” data regarding BRS in the setting of AMI and helped determine patients for whom BRS implantation is suitable.

## Conclusions

The implantation of BRS and DP-EES showed similar 1-year efficacy and safety profiles in the setting of AMI when intravascular image-guided BRS implantation, especially OCT, is performed in young patients with low-risk profiles.

## Supporting information

S1 Data (XLSX)
